# Personal

**Published:** 1863-02

**Authors:** 


Personal.—Dr. W. W. Potter, formerly Assistant Surgeon 49th N. Y. V. has recently received appointment as *Surgeon* to the 57th N. Y. V. Dr. Potter has served with the 49th N. Y. for the last eighteen months, and will bring a very good and well earned reputation to the field of his new appointment. Dr. P. is from Colesville, and a graduate of the University of Buffalo. He has had a very varied experience during his service; was taken prisoner on the 29th June and retained three weeks, (long enough to get a glimpse of "rebeldom,") and was released with the first prisoners who were exchanged under the cartel.

The homœopaths have again appealed to Congress to experiment with infinitesimals in the treatment of sick soldiers, and the Committee to whom their petition was referred has asked to be discharged from its consideration. It was not surprising that homœopathy should clamor for recognition while a homœopathic commanding General was its patron: but now that he is superseded, and the medical staff is being thoroughly purged of all its incompetents, it is remarkable that the advocates of this delusion should presume to ask admission to the medical service. They would, if admitted, meet with a summary dismissal.—*Med. Times.*

Dr. Morton, the alleged discoverer of the application of ether to practice, has again appealed to Congress for Compensation for the use of this agent in the Army. It will be remembered that a similar claim was pressed through both houses several years ago, but failed to obtain the President's signature. Dr. Morton subsequently appealed to the medical profession for relief, and large sums of money were freely subscribed in Boston and New York towards a national testimonial. Recently the patent expired, and a renewal was refused. Dr. M. then instituted a suit against the N. Y. Eye Infirmary, but failed to establish the right of prosecution. He now returns to Congress, and renews his petition for compensation. If he was ever entitled to anything the Government should be the donor.—*Medical Times.*

